# The safety and efficacy of delayed surgery by simulating clinical progression of observable papillary thyroid microcarcinoma: a retrospective analysis of 524 patients from a single medical center

**DOI:** 10.3389/fonc.2023.1046014

**Published:** 2023-10-10

**Authors:** Liuhong Shi, Kehao Le, Haiou Qi, Yibing Feng, Liang Zhou, Jianbiao Wang, Lei Xie

**Affiliations:** ^1^Department of Head and Neck Surgery, Affiliated to Sir Run Run Shaw Hospital, Zhejiang University School of Medicine, Hangzhou, Zhejiang, China; ^2^Department of Nursing, Affiliated to Sir Run Run Shaw Hospital, Zhejiang University School of Medicine, Hangzhou, Zhejiang, China; ^3^Department of Second Surgery, Longyou County People’s Hospital, Sir Run Run Shaw Hospital, Quzhou, Zhejiang, China

**Keywords:** papillary thyroid carcinoma, microcarcinoma, active surveillance, lymph node, response to therapy

## Abstract

**Objective:**

When active surveillance (AS) is developed in the patients with low-risk papillary thyroid microcarcinoma (PTMC), a medical center needs to ensure the delayed operation that is caused by PTMC clinical progression to have the same prognosis as that of immediate operation. The objective of this study was to investigate the efficacy of delayed surgery by simulating clinical progression (tumor size enlargement and appearance of lymph node metastasis) of PTMCs with AS in a single medical center.

**Methods:**

We retrospectively analyzed the response to therapy in 317 papillary thyroid carcinoma patients treated with total thyroidectomy and post-operative radioactive iodine ablation. They were classified into three groups according to tumor size (group A ≤0.5 cm; group B >0.5 cm and ≤1 cm; group C >1 cm and ≤1.5 cm) or two groups according to the presence (cN1) or absence (cN0) of the clinical lymph node (LN) metastasis. Groups C and cN1 were regarded as simulated clinical progression of observational PTMC and the operation for them was assumed to be “delayed surgery”. However, Groups A, B and cN0 were regarded as no clinical progression and the operation for them was considered as immediate surgery.

**Results:**

There were no significantly differences in excellent response to therapy and recurrence-free survival not only among the group A, B and C, but also between the group cN0 and cN1. In other words, these insignificant differences were found between immediate and simulated “delayed” surgeries.

**Conclusion:**

For the PTMC patients suitable for AS, the oncological outcomes were also excellent even if surgery was delayed until after the presence of clinical progression, according to our clinical simulation. Furthermore, we consider that it was feasible for medical centers to assess the ability to implement AS for PTMC patients by retrospectively analyzing their own previous clinical data using the described simulation.

## Introduction

Papillary thyroid carcinoma (PTC) is the most common endocrine malignancy, and it usually has an indolent biological nature. When the cancer measures ≤10 mm in its largest diameter, it is called a papillary thyroid microcarcinoma (PTMC). A rapid increase in the incidence of PTC has been reported in the past several decades in many countries, and approximately 50% of PTCs are PTMCs (1). However, a majority of PTMCs are occult and detected because of overscreening in the healthy population. On this basis, in 1993, Professor Miyauchi of Kuma Hospital in Japan proposed that low-risk PTMC patients could be followed with active surveillance (AS) rather than immediately undergo surgery; their later study showed that the incidence rates of size enlargement, novel appearance of node metastasis, and progression to clinical disease in 1,235 PTMC patients were 8.0%, 3.8%, and 6.8% over a 10-year observation period, respectively (2). Therefore, presently AS is the first-line management of low-risk PTMC patients at Kuma Hospital (3).

Although AS is an option for PTMC patients, as stated in the 2015 guidelines of the American Thyroid Association (ATA) (4), questions remain as to how to identify low-risk patients, how to deal with 30%~40% subclinical metastasis in central neck lymph nodes (LNs), and how to relieve the anxiety of patients and their families during the observation period. The major concerns are, for PTMC patients under AS, whether the prognosis of immediate operation is the same as that of delayed surgery due to clinical progression, whether the medical center has an ability to reach, and how to assess this ability. However, few studies in this area have been published.

The recurrence risk estimate is one of the most important ways of evaluating prognosis in PTC patients; therefore, a response to therapy system was proposed in the 2015 ATA guidelines (4). According to the clinical data obtained from imaging examinations and biochemical and cytopathological findings, the postoperative clinical responses of PTC patients to therapy are described as follows: an excellent response, a biochemical incomplete response, a structural incomplete response, and an indeterminate response. In this study, we retrospectively analyzed the response to therapy in PTMC patients initially eligible for AS, with different size tumors and with or without LN metastasis, to investigate the efficacy of delayed surgery by simulating clinical progression (tumor size enlargement and appearance of LN metastasis) in a single medical center.

## Materials and methods

### Patients

**Figure 1:**
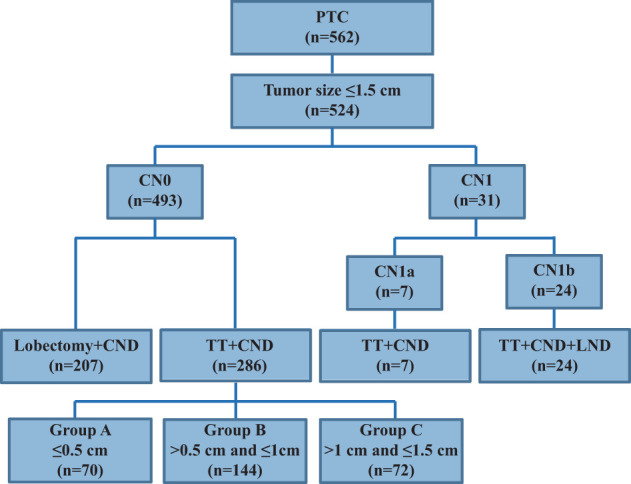
Flow diagram of included papillary thyroid carcinoma patients.

Between January 2013 and December 2015, 562 PTC patients underwent unilateral lobectomy or total thyroidectomy (TT) and central neck dissection (CND), with or without lateral neck dissection (LND), as an initial surgical treatment at our hospital (**Figure 1**). Among them, 524 patients with a primary tumor size ≤1.5 cm and who had undergone thyroidectomy were enrolled in this study. Exclusion criteria were as follows: no PTC diagnosis, aggressive histology (e.g. tall cell, hobnail variant, columnar cell carcinoma), distant metastasis, extrathyroidal extension to nearby organs (e.g. trachea or esophagus), a poorly differentiated PTC (diagnostic criteria were on the basis of the Turin consensus proposal) (5), a coexisting malignancy, a thyroid lobectomy, a tumor size >1.5 cm, and no radioactive iodine (RAI) ablation. Finally, 317 patients met the selection criteria and included 286 cN0 and 31 cN1 patients. The current definition of “clinically apparent” LN metastasis (cN1 disease) includes any metastatic LN identified by palpation or imaging either before initial surgery or intraoperatively (6). When a suspicious LN appeared at level VI or II-IV, it was defined as cN1a or cN1b, respectively. Accordingly, patients without cN1 disease were defined as cN0. Therefore, 286 cN0 and seven cN1a patients underwent TT+CND, and 24 cN1b patients underwent TT+CND+LND. These 286 cN0 patients were enrolled to analyze the clinicopathologic characteristics associated with the size of their primary tumors and to compare them with 31 cN1 patients to analyze the clinicopathologic differences caused by clinically involved neck LN metastasis. After surgery, a histological diagnosis was confirmed by two experienced pathologists from the Department of Pathology. The study was approved by the Ethics Committee of the Affiliated Sir Run Run Shaw Hospital, Zhejiang University School of Medicine, and all enrolled patients provided informed consent.

### Treatment protocol

The indication of TT was strictly in accordance with the 2009 ATA guidelines (7). Shi L et al. described the indications of CND and modified LND (8). RAI remnant ablation was performed postoperatively in all patients with TT according to the 2009 ATA guidelines (7).

### Assessment of treatment response

The response to the therapy re-staging system has been designed for the follow-up of PTC patients in the 2015 ATA guidelines. According to biochemical, imaging, and cytopathologic findings, for patients treated with TT and RAI remnant ablation, there were four response-to-therapy categories, including excellent, biochemical incomplete, structural incomplete, and indeterminate responses (4). In this study, we rearranged them into two categories consisting of excellent response and non-excellent response. The latter included biochemical incomplete, structural incomplete, and indeterminate responses. According to the ATA guidelines, the recurrence rate for the excellent response group is 1%–4%, which is much lower compared with that for other groups (biochemical incomplete response: 20% develop structural disease, structural incomplete response: 50%–85% continue to have persistent disease despite additional therapy, and indeterminate response: 15%–20% will have structural disease) (4).

All PTC patients were followed after completion of RAI remnant ablation. Routine neck ultrasound examinations and measurement of serum thyroglobulin (Tg) and anti-Tg antibodies were performed in a state of thyroid-stimulating hormone suppression every 3 months in the first year and every 6–12 months thereafter. When biochemical or structural incomplete responses occurred, more frequent follow-up was recommended, and additional examinations (e.g. computed tomography scan and fine needle aspiration) were proposed. Postoperative follow-up periods for the 317 patients ranged from 35–75 months (median follow-up time, 57 months), and the assessment of response to therapy was on the basis of the latest examination results. Serum Tg (normal range, 1.15–35.00 ng/mL) and Tg antibodies (normal range, 0–4.11 IU/mL) were measured using an electrochemiluminescence immunoassay in an Abbott Aeroset^®^ automated instrument analyzer (Toshiba Medical Systems, Tochigi, Japan) (9–11). When TgAb exceeded the upper limit of its normal value, the value of TgAb was considered as positive.

### Study design

The patients were classified into three groups according to tumor size (group A ≤0.5 cm; group B >0.5 cm and ≤1 cm; group C >1 cm and ≤1.5 cm) or two groups according to the presence (cN1) or absence (cN0) of clinical LN metastasis. Groups C and cN1 were regarded as simulated clinical progression of observable PTMC and the operation for them was assumed to be “delayed surgery”, whereas Groups A, B and cN0 were regarded as no clinical progression and the operation for them was considered as immediate surgery. The response-to-therapy and recurrence-free survival (RFS) were analyzed among these groups in order to compare the safety and effectiveness between immediate and simulated “delayed” surgeries.

### Statistical analysis

Continuous variables and categorical variables were determined using the Mann–Whitney U and chi-squared tests (including Fisher’s exact probability tests, if needed), respectively. The results were presented as medians with range and numbers with percentages. Univariate logistic regression analyses were performed on gender, age, tumor size, tumor multifocality, ETE, chronic lymphocytic thyroiditis (CLT), and N stage. The variables exhibiting p<0.05 in univariate analysis were then selected and analyzed using multivariate logistic regression analysis. The results are represented as odds ratios (ORs) with 95% confidence intervals (CIs). For all analyses, two-sided tests were employed, and differences with p<0.05 were regarded as statistically significant. Statistical analyses were performed using SPSS software (version 23.0, Inc., Chicago, IL, USA).

## Results

### Characteristics of PTC patients who underwent TT

It is shown in **Figure 1** that 524 PTC patients with tumor sizes ≤1.5 cm were enrolled, but 207 cN0-PTC patients who underwent lobectomy and CND were excluded from our study. The remaining 317 PTC patients who underwent TT and CND or together with LND were enrolled and included 286 cN0 and 31 cN1 cases.

The clinicopathological characteristics of the 317 PTC patients are summarized in **Table 1**; **Figure 1**. The median age of the cohort was 43 years (range, 13–72 years). Most patients were women (77.6%), and most were less than 55 years old (82.6%). Patients were grouped according to tumor size: ≤0.5 cm (22.7%), >0.5 cm and ≤1 cm (51.1%), >1 cm and ≤1.5 cm (26.2%). Multifocality, CLT, and ETE were found in 58.0% (184/317), 26.5% (84/317), and 30.3% (96/317) of patients, respectively. The clinical N stage included 286 cN0 (90.2%), seven cN1a (2.2%), and 24 cN1b (7.6%) patients, whereas the pathological N stage included 155 pN0 (48.9%), 138 pN1a (43.5%), and 24 pN1b (7.6%) patients. An excellent response to primary therapy was observed in 249 patients (78.6%). According to ATA risk stratification, there were 155 (48.9%), 82 (25.9%), and 80 (25.2%) patients in the low, intermediate, and high-risk categories, respectively.

**Table 1:** Characteristics of 317 PTC patients with total thyroidectomy.

Characteristics	Total (n=317)
Gender	
Male (22.4%)	71
Female (77.6%)	246
Age of diagnosis (years)	
Median (range), year	43 (13-72)
<55 years (82.6%)	262
≥55 years (17.4%)	55
Primary tumor size (cm)	
≤0.5 (22.7%)	72
>0.5 and ≤1 (51.1%)	162
>1 and ≤1.5 (26.2%)	83
Multifocality	
Absent (42.0%)	133
Present (58.0%)	184
CLT	
Absent (73.5%)	233
Present (26.5%)	84
ETE	
Absent (69.7%)	221
Present (30.3%)	96
Postoperative complications	
Absent (86.7%)	275
Temporary VCP (1.6%)	5
Permanent VCP (0.0%)	0
Temporary Hypo-PT (11.7%)	37
Permanent Hypo-PT (0.0%)	0
Clinical Node stage ^a^	
cN0 (90.2%)	286
cN1a (2.2%)	7
cN1b (7.6%)	24
Pathological Node stage ^a^	
pN0 (48.9%)	155
pN1a (43.5%)	138
pN1b (7.6%)	24
ATA response-to-therapy categories ^b^	
Excellent Response (78.6%)	249
Biochemical Incomplete Response (0.3%)	1
Structural Incomplete Response (2.5%)	8^d^
Indeterminate Response (18.6%)	59
ATA Risk stratification ^c^	
Low Risk (48.9%)	155
Intermediate Risk (25.9%)	82
High Risk (25.2%)	80

a Tumor, node, metastasis (TNM) classification system established by the American Joint Commission on Cancer (AJCC; 2010, 7th edition).

b Response to therapy according to the 2015 American Thyroid Association (ATA) management guidelines for differentiated thyroid cancer patients treated with total thyroidectomy and RAI remnant ablation.

c Risk stratification system according to the 2015 American Thyroid Association (ATA) management guidelines for adult patients with thyroid nodules and differentiated thyroid cancer.

CLT, chronic lymphocytic thyroiditis; ETE, extra-thyroidal extension.

d Among these eight patients, recurrence was confirmed by reoperation in four patients, while in other four patients, recurrence was highly suspicious by ultrasound examination but was not proven by surgery or biopsy. In our study, recurrence was diagnosed if the lesion recurred after 12 months of no evidence of disease, according to the guidelines of the Chinese Society of Clinical Oncology (12).

### Patient clinicopathologic features associated with the primary tumor size of cN0-PTC

There were 286 cN0-PTC patients who were divided into three groups according to their primary tumor size: group A ≤0.5 cm (n=70), group B >0.5 cm and ≤1 cm (n=144), and group C >1 cm and ≤1.5 cm (n=72). As shown in **Table 2**, P1 represents the statistical difference between group A and group B, and P2 represents the statistical difference between group B and group C. Because group B was used in the statistical analysis twice, we subsequently used Bonferroni correction and defined P<0.025 (P<0.05/2) as statistically different.

**Table 2:** Characteristics of pN1a and pN0 patients according to primary tumor size.

Characteristics	Primary tumor size (cm) (n=286)			*P1*	*P2*
	Group A ≤0.5 (n=70)	Group B >0.5 and ≤1 (n=144)	Group C >1 and ≤1.5 (n=72)		
Gender					
Male	11 (15.7%)	26 (18.1%)	19 (26.4%)	0.671	0.155
Female	59 (84.3%)	118 (81.9%)	53 (73.6%)		
Age of diagnosis (years)					
Median (range), year	43 (23-69)	43 (23-72)	45 (13-66)	0.836	0.389
<55 years	55 (78.6%)	119 (82.6%)	59 (81.9%)	0.474	0.899
≥55 years	15 (21.4%)	25 (17.4%)	13 (18.1%)		
Multifocality					
Absent	27 (38.6%)	47 (32.6%)	48 (66.7%)	0.393	<0.001
Present	43 (61.4%)	97 (67.4%)	24 (33.3%)		
Extrathyroidal extension					
Absent	58 (82.9%)	101 (70.1%)	44 (61.1%)	0.046	0.183
Present	12 (17.1%)	43 (29.9%)	28 (38.9%)		
CLT					
Absent	47 (67.1%)	106 (73.6%)	55 (76.4%)	0.325	0.659
Present	23 (32.9%)	38 (26.4%)	17 (23.6%)		
Central lymph node metastasis					
Absent (N0)	54 (77.1%)	74 (51.4%)	28 (38.9%)	< 0.001	0.083
Present (N1a)	16 (22.9%)	70 (48.6%)	44 (61.1%)		
Number of CmLN					
Median (range)	0 (0-5)	0 (0-18)	1 (0-8)	< 0.001	0.105
≤5	70 (100%)	139 (96.5%)	67 (93.1%)	0.273 ^#^	0.423^#^
>5	0 (0%)	5 (3.5%)	5 (6.9%)		
Temporary VCP ^a^					
Absent	70 (100%)	142 (98.6%)	70 (97.2%)	1.000^*^	0.858^#^
Present	0 (0%)	2 (1.4%)	2 (2.8%)		
Temporary Hypo-PT ^b^					
Absent	59 (84.3%)	127 (88.2%)	63 (87.5%)	0.426	0.882
Present	11 (15.7%)	17 (11.8%)	9 (12.5%)		
Response-to-therapy ^c^					
Excellent Response	54 (77.1%)	118 (81.9%)	55 (76.4%)	0.407	0.335
Non- Excellent Response	16 (22.9%)	26 (18.1%)	17 (23.6%)		

P1: P value of statistical analysis between group A and group B.

P2: P value of statistical analysis between group B and group C.

a No permanent VCP was found in any cases.

b No permanent hypoPT was found in any cases.

c Response to therapy on the basis of the 2015 American Thyroid Association (ATA) management guidelines for differentiated thyroid cancer patients treated with total thyroidectomy and RAI remnant ablation.

^#^Continuity correction^b^.

^*^Fisher’s exact test.

According to the Bonferroni correction test, P<0.025 (0.05/2) is defined as statistically different.

Comparison of the postoperative pathologic results revealed that most clinicopathologic factors were not statistically different (P>0.025) between group A and group B (P1) or group B and group C (P2). These factors were gender, age, multifocality, ETE, CLT, temporary vocal cord paralysis (VCP), temporary hypoparathyroidism (hypoPT), and response to therapy. To describe the features of the mLNs, a statistical analysis was performed for the presence and number of central mLNs (CmLNs). We found that these two factors were significantly different between group A and group B (P1<0.001, both) but not different between group B and group C (P2 = 0.083, P2 = 0.105, respectively). According to the 2015 ATA guidelines, patients with mLNs ≤5 belong to the low-risk category. Therefore, we regarded mLNs ≤5 as an index, and we found no difference among these three groups (P1 = 0.273, P2 = 0.423). Furthermore, no significant difference was presented in staging of the response to therapy among these three groups. Therefore, it could be seen that the incidence and the number of mLNs in the central area were increasing when the tumor increased in size, especially from 0.5–1 cm, and that the incidence of excellent response was the same in three groups with different size primary tumors ranging from 0–1.5 cm.

### A comparison of clinicopathologic features between cN0-PTC and cN1-PTC patients with tumors ≤1.5 cm

We divided 317 patients with primary lesions ≤1.5 cm into two groups according to whether their neck LN was clinically metastatic: cN0-PTC (n=286) and cN1-PTC (n=31). A comparison of the clinicopathologic features between the two groups is shown in **Table 3**.

**Table 3:** Characteristics of PTC patients with tumors ≤1.5 cm according to cN0 and cN1.

Characteristics	cN0 (n=286)	cN1 (n=31)	*P*
Gender			
Male	56 (19.6%)	15 (48.4%)	<0.001
Female	230 (80.4%)	16 (51.6%)	
Age of diagnosis (years)			
Median (range), year	43 (13-72)	37 (20-58)	0.004
<55 years	233 (81.5%)	29 (93.5%)	0.092
≥55 years	53 (18.5%)	2 (6.5%)	
Tumor size (cm)	0.7 (0.2-1.5)	1.0 (0.5-1.5)	0.002
≤0.5	70 (24.5%)	2 (6.5%)	<0.001
>0.5 and ≤1	144 (50.3%)	18 (58.1%)	
>1 and ≤1.5	72 (25.2%)	11 (35.4%)	
Multifocality			
Absent	122 (42.7%)	11 (35.5%)	0.442
Present	164 (57.3%)	20 (64.5%)	
Extrathyroidal extension			
Absent	203 (71.0%)	18 (58.1%)	0.137
Present	83 (29.0%)	13 (41.9%)	
CLT			
Absent	208 (72.7%)	25 (80.6%)	0.343
Present	78 (27.3%)	6 (19.4%)	
Temporary VCP ^a^			
Absent	282 (98.6%)	30 (96.8%)	0.404
Present	4 (1.4%)	1 (3.2%)	
Temporary Hypo-PT ^b^			
Absent	249 (87.1%)	31 (100%)	0.066
Present	37 (12.9%)	0 (0%)	
Response-to-therapy ^c^			
Excellent Response	227 (79.4%)	22 (71.0%)	0.279
Non- Excellent Response	59 (20.6%)	9 (29.0%)	

a No permanent VCP was found in any cases.

b No permanent hypoPT was found in any cases.

c Response to therapy based on the 2015 American Thyroid Association (ATA) management guidelines for differentiated thyroid cancer patients treated with total thyroidectomy and RAI remnant ablation.

The results showed that cN1-PTC patients comprised significantly more males (48.4% vs. 19.6%, P<0.001), younger ages (37 vs. 43, p=0.004), and larger sized tumors (1.0 cm vs. 0.7 cm, P=0.002) than cN0 patients. There was no statistical difference between the two groups regarding multifocality, ETE, CLT, temporary VCP, temporary hypoPT, or response to therapy (P>0.05, all). Therefore, cN1 was associated with larger tumor size, but was unassociated with other aggressive characteristics, and the incidence of excellent response was the same whether PTMC patients had clinical metastasis or not.

### Risk factors for non-excellent response to therapy in PTC patients with TT

Risk factors for non-excellent response to therapy was analyzed in 317 PTC patients (286 cN0 and 31 cN1). As shown in **Table 4**, univariate analysis indicated that CLT (<0.001) and >5 pathological CmLNs (p=0.002) significantly increased the risk of classification into the non-excellent response to therapy category. Furthermore, these two factors were independent variables for response to therapy in multivariate analysis (p<0.001). Therefore, we determined that CLT (absent vs. present) and the number of pathological CmLNs (≤5 vs. >5) represented independent risk factors for predicting clinical outcome, rather than tumor size or cN1.

**Table 4:** Relationships between clinicopathologic variables and non-excellent response to therapy in PTC patients with total thyroidectomy.

Characteristics	Univariate		Multivariate	
	OR (95% CI)	*p*	OR (95% CI)	*p*
Gender (Male vs Female)	1.882 (0.906-3.907)	0.090		
Age of diagnosis (years) (<55 vs ≥55)	0.573 (0.257-1.279)	0.174		
Primary tumor size (cm) (≤1 vs >1)	1.348 (0.748-2.429)	0.321		
Multifocality (Absent vs Present)	1.317 (0.758-2.290)	0.329		
ETE (Absent vs Present)	0.587 (0.312-1.104)	0.098		
CLT (Absent vs Present)	5.130 (2.892-9.099)	<0.001	6.179 (3.365-11.343)	<0.001
Clinical N stage (cN0 vs cN1)	1.574 (0.689-3.598)	0.282		
Pathological N stage (pN0 vs pN1)	1.400 (0.815-2.405)	0.223		
Number of pathological CmLN (≤5 vs >5)	3.582 (1.571-8.167)	0.002	5.617 (2.292-13.768)	<0.001

CLT, chronic lymphocytic thyroiditis; ETE, extra-thyroidal extension; CmLN, central neck metastatic lymph node.

### Recurrence-free survival according to tumor size and clinical N stage

**Figure 2:**
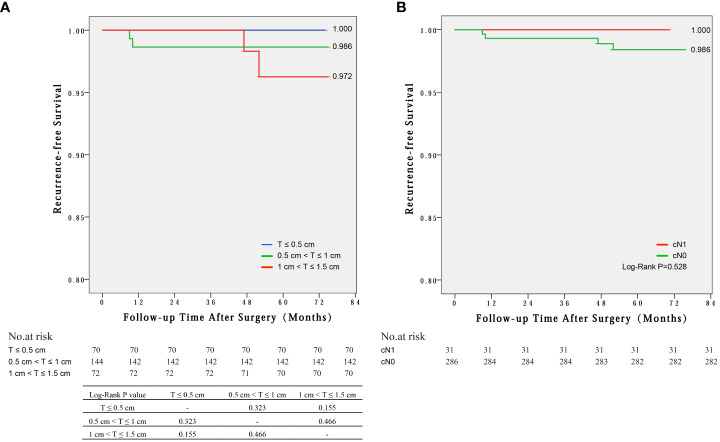
Recurrence-free survival by Kaplan-Meier curves for different groups: **(A)** Tumor sizes of 1~1.5cm, 0.5~1cm and ≤0.5 cm, **(B)** cN1 and cN0 groups.

Four cases of disease recurrence were identified during the median follow-up period of 57 months (range 35–75 months) across all PTC patients. The mean time of recurrence was 29.5 months. The RFS were not significantly different among the three groups of 1~1.5cm, 0.5~1cm and ≤0.5 cm (97.2% vs. 98.6% vs. 100%, log-rank P>0.05) (**Figure 2A**) and between cN1 and cN0 groups (100% vs. 98.6%, log-rank P=0.528) (**Figure 2B**).

## Discussion

At Kuma Hospital, the indications for surgery in low-risk PTMC patients under AS are tumor enlargement ≥3 mm, tumors that are 1.2 cm in diameter, or LN metastasis (called clinical progression) (13). Therefore, we used our data to simulate clinical progression and designed this study in two parts. Part one involved dividing the cN0 patients into three groups according to tumor size (≤0.5 cm, 0.5~1 cm and 1~1.5 cm) to simulate tumor size increase. We found the incidence and the number of mLNs in the central area were increasing when the tumor increased in size, especially from 0.5–1 cm, but there was no significant difference in the incidences of excellent response and RFS among these three groups. Part two involved setting up two groups: cN0 and cN1 to simulate metastatic LN appearance. We found cN1 was associated with larger tumor size, but was unassociated with other aggressive characteristics, and the incidences of excellent response and RFS were the same whether PTMC patients had clinical metastasis or not. Additionally, we determined that CLT (absent vs. present) and the number of pathological CmLNs (≤5 vs. >5) represented independent risk factors for predicting clinical outcomes, rather than tumor size or clinical lymph node metastasis. If we set tumors with 1~1.5cm in size or cN1 as clinical progression of observable PTMC, then their corresponding surgeries can be considered as simulated “delayed surgery”. Our results suggested, therefore, that the oncologic outcome of “delayed surgery” for PTMC with AS showing clinical progression was as good as that of immediate operation.

Woolner and his colleagues from the Mayo Clinic were the first scientists to coin the term occult papillary carcinoma for PTCs ≤1.5 cm in size in 1960 (14). Their study showed that occult papillary carcinoma patients had a good prognosis on the basis of 30 years of follow-up of 140 cases. In 1989, the World Health Organization introduced the term papillary microcarcinoma to replace the term occult papillary carcinoma, and they defined papillary microcarcinomas as being PTCs ≤1 cm in diameter (15). PTMC was regarded as an important variant of PTC because of its low malignancy and exceptionally rare distant metastasis: 6%~35% frequency as incidental findings in autopsy studies, and increasing frequency in life by modern methods of investigation. Hence the size of low-risk PTMCs suitable for observation generally range between 1 cm and 1.5 cm. However, the Chinese Association of Thyroid Oncology (CATO) suggests that the size of observed low-risk PTMCs should be not greater than 0.5 cm (16). CATO’s view is on the basis of a contrastive study of two screening criteria for AS in 1,001 low-risk PTMC patients. Compared with that in the Kuma low-risk PTMC group (≤1 cm), Qian et al. found a lower incidence of multifocal lesions, ETEs, central LN metastasis, progression rates, and prolonged DFS in the CATO low-risk PTMC group (≤0.5 cm). Therefore, in our study on PTMC, the PTC patients with primary tumors ≤1.5 cm were chosen and were divided into three groups according to tumor size, and the tumors sized 1 to 1.5 cm were assumed to be clinical progression of observable PTMC.

The emergence of “delayed surgery” is accompanied by AS practiced for PTMC, so it has been well-known. However, in fact, there are few relative articles found and the long-term clinical outcomes after delayed surgery remain unclear (17). Korean scholars did an interesting study. A total of 2863 PTMC patients were assigned into three groups due to a delay period of ≤6 months, 6–12 months, and >12 months. They found that there were no significant differences in the development of structural recurrent/persistent disease and disease-free survival among the groups (18). Actually, they simulated a situation that patients with PTMC could be observed for a period of time before operation. The reason of their delayed operations was not clinical progression of PTMC, so it had nothing to do with “delayed operation” for the PTMCs with simulation clinical progression in our article. In addition, “delayed surgery” was really mentioned in some articles, but it was often taken in one stroke (19, 20).

Miyauchi and his colleagues found that rapid or slow growth happened in approximately one quarter of PTMCs (21), which will be likely switched to delayed surgery. Therefore, another issue facing surgeons is whether delaying surgery will increase surgical complications or not. In the study of Oda et al., they analyzed the incidence of unfavorable events in PTMC patients between AS and immediate surgery groups, and they showed that the oncological outcomes of the immediate surgery and AS groups were similarly excellent, but the incidence of unfavorable events were higher in the immediate surgery group (20). However, by further analyzing their data on surgical complications in all patients who were operated on, we found that the incidences of temporary VCP and hypoPT in the delayed surgery group were actually higher than in the immediate surgery group, although the incidences of permanent ones were similar between the two groups. This result from their data is logical, because larger or more lesions must also increase surgical difficulty. As for our study, there were no significant differences in the incidence of surgical complications among the groups (see **Table 2**). However, we also emphasized that certain PTMCs that occur in anatomically sensitive sites should be identified and treated surgically, such as those located in the area adjacent to the entrance of the recurrent laryngeal nerve to the larynx, the so-called “danger triangle” (22).

Although still controversial, more and more endocrinologists and some thyroid surgeons and medical teams are beginning to use AS in low-risk PTMC patients. Therefore, the choice of AS is a very serious issue not only for the patients and their families, but also for doctors, especially in China (23, 24). Prescribing AS is a decision that involves considering three aspects, namely inherent tumor characteristics, patient characteristics, and medical team characteristics. Among these three aspects, we think that an experienced medical group is a vital factor in performing AS smoothly. It should not only include multidisciplinary cooperation, an excellent sonographer, and an active management of patients and their data, but also the ability to retrospectively analyze the medical group’s own patients previously surgically treated, like in this study, to answer the question “whether the therapeutic result of delayed surgery is the same as that of immediate surgery in my medical center”. We would suggest that only if the result of its data analysis is acceptable should the medical team propose AS for low-risk PTMC patients.

This study had several limitations. First, there were not many relapsed cases in our consecutive patients from 2013–2015, and therefore we had to choose the index of response to therapy to evaluate the therapeutic effect. Because this assessment system is usually performed in PTC patients treated with TT and RAI ablation in a majority of published studies (25–27). Therefore, in our study we just enrolled the PTMC patients treated with TT and RAI ablation, and we excluded those with thyroid lobectomy. Second, the treatment for our PTC patients from 2013–2015 was performed according to the 2009 ATA guidelines. If we refer to the 2015 ATA guidelines, our disease management at that time must be regarded as over-treatment, and thyroid lobectomy should have been enough for the majority of them. Presently, we cannot use our data to answer the question of whether thyroid lobectomy performed after the presence of clinical progression is also feasible. Third, generally speaking, the evolution of papillary thyroid carcinoma is a very slow process and a patient with the same tumor size may not have the same tumor evolution process. However, a tumor with 1cm in size must have grown from a tumor with 0.5cm in size, and a tumor with lymphatic metastasis must have developed from no metastasis. Therefore, this study was done by simulating clinical progression of PTMCs with AS. Groups C and cN1 were regarded as simulated clinical progression of observable PTMC and the operation for them was assumed to be “delayed surgery”, whereas, Groups A, B and cN0 were regarded as no clinical progression and the operation for them was considered as immediate surgery. Forth, there were only 317 consecutive patients enrolled from 2013-2015 with a relatively short follow-up period. Therefore, additional studies with longer follow-up and multicenter data are needed.

To some extent, this study reflected that the oncological outcomes were also excellent even if surgery was delayed until after the presence of clinical progression (tumor size enlargement and appearance of LN metastasis), according to our clinical simulation. Furthermore, we considered that it was feasible for medical centers to assess the ability to implement AS in PTMC patients by retrospectively analyzing their own previous clinical data in the described simulation.

## Data availability statement

The raw data supporting the conclusions of this article will be made available by the authors, without undue reservation.

## Ethics statement

The study was approved by the Ethics Committee of the Affiliated Sir Run Run Shaw Hospital, Zhejiang University School of Medicine, and all enrolled patients provided informed consent.

## Author contributions

LS and LX designed the study and confirm the authenticity and legitimacy of all raw data. LZ, JW and KL performed the data collection and analysis. LS interpreted the results. HQ and YF performed the follow‐up plan and collected the data from patients. LX supervised the project. All authors read and approved the final manuscript.
